# IgG Fc engineering to modulate antibody effector functions

**DOI:** 10.1007/s13238-017-0473-8

**Published:** 2017-10-06

**Authors:** Xinhua Wang, Mary Mathieu, Randall J. Brezski

**Affiliations:** Genentech, Antibody Engineering, South San Francisco, CA 94080 USA

**Keywords:** antibody-dependent cell-mediated cytotoxicity, antibody-dependent cellular phagocytosis, complement-dependent cytotoxicity, Fc engineering, Fc gamma receptor, monoclonal antibody, neonatal Fc receptor

## Abstract

Therapeutic monoclonal antibodies are among the most effective biotherapeutics to date. An important aspect of antibodies is their ability to bind antigen while at the same time recruit immune effector functions. The majority of approved recombinant monoclonal antibody therapies are of the human IgG1 subclass, which can engage both humoral and cellular components of the immune system. The wealth of information generated about antibodies has afforded investigators the ability to molecularly engineer antibodies to modulate effector functions. Here, we review various antibody engineering efforts intended to improve efficacy and safety relative to the human IgG isotype. Further, we will discuss proposed mechanisms by which engineering approaches led to modified interactions with immune components and provide examples of clinical studies using next generation antibodies.

## INTRODUCTION

Antibodies are a critical component of the immune response, and the advent of the monoclonal antibody (mAb) technology has afforded the biotechnology and pharmaceutical industries the ability to develop antibodies as drugs. The success of mAb-based therapies is based on their safety, selectivity, diversity, solubility, tolerability, stability, and long circulating half-life. This success is further exemplified by the fact that there are currently over 50 investigational antibodies undergoing evaluation in late-stage clinical trials (Reichert, 2017) and approved antibodies generate an annual market of over $60 billion (Ecker et al., 2015).

**Figure 1:**
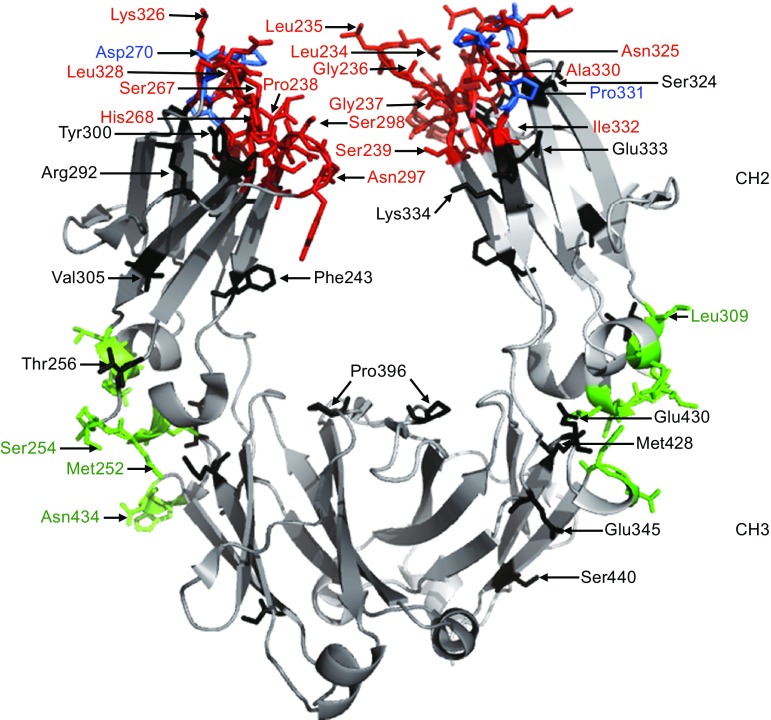
**The Fc domain of human IgG1 is shown (PDBID:3AVE)**. The positions of amino acids described in Table 1 are shown. Key sequence motifs for FcRn interactions are shown in green (L251-S254; L309-Q311; N434-H435) (Oganesyan et al., 2014). Amino acids 5 angstroms proximal to the FcγR:Fc interface for FcγRI (PDBID:4W4O) (Kiyoshi et al., 2015), FcγRIIa (PDBID:3RY6) (Ramsland et al., 2011), FcγRIIb (PDBID:3WJJ) (Mimoto et al., 2013b), and FcγRIIIb (PDBID:1E4K) (Sondermann et al., 2000) as determined with PyMOL are shown in red (P232-S239; D265-D270; Y296-T299; N325-I332). The amino acids that are critical for interactions with C1q are shown in blue (D270, K322, P329, P331) (Idusogie et al., 2000). Amino acids depicted in black were not identified as critical for interactions with FγRs, FcRn, or C1q. Figure 1 was generated with PyMOL.

To date, all of the currently approved antibodies are of the IgG isotype, which is further divided into four subclasses in humans (i.e., IgG1, IgG2, IgG3, and IgG4) (Brezski and Georgiou, 2016; Lefranc, 2001). The structure of an IgG antibody is comprised of two antigen-binding Fab arms linked to a single Fc domain via the hinge region. This structural arrangement allows antibodies to link bound antigen with humoral and cellular components of the immune system. Engagement of the humoral immune response is governed, in large part, by interactions with C1q and the initiation of a series of proteolytic events known as the complement cascade (Meyer et al., 2014). The cellular immune response occurs mostly due to the interactions between the antibody and Fc gamma receptors (FcγRs). There are 5 activating FcγRs: the high affinity FcγRI (HGNC:3613) that can bind to monovalent antibody, and the lower affinity FcγRIIa (HGNC:3616) and IIc (HGNC:15626), and FcγRIIIa (HGNC:3619) and IIIb (HGNC:3620) that require avidity-based interactions. There is one inhibitory receptor: FcγRIIb (HGNC:3618). Intracellular signaling through the activating receptors is modulated through the phosphorylation of immunoreceptor tyrosine-based activation motifs (ITAMs), which leads to effector functions such as antibody-dependent cell-mediated cytotoxicity (ADCC), antibody-dependent cellular phagocytosis (ADCP), and inflammation via the induction of cytokine secretion. In contrast, intracellular signaling through the inhibitory FcγRIIb is modulated through the phosphorylation of immunoreceptor tyrosine-based inhibitory motifs (ITIMs), which recruit phosphatases that counter-balance activating signaling pathways (Nimmerjahn and Ravetch, 2008). Antibody interactions with FcγRs and C1q are dependent on the hinge and proximal CH2 amino acid sequence as well as glycosylation at the conserved amino acid N297 (EU numbering (Edelman et al., 1969)) in the CH2 region (Fig. 1).

Although many antibody-based therapies have had clinical and commercial success, antibody-based therapeutics are often only effective in a subset of patients. Therefore, considerable effort has been put into fine-tuning the properties of antibodies in attempts to achieve efficacy in larger patient populations. Among the many efforts, one area of focus has been modulating the ability of antibodies to interact with the humoral and cellular components of the immune system through a process known as Fc engineering (Carter, 2006; Presta, 2008; Strohl, 2011). The purpose of this review is to detail Fc engineering efforts that modulate effector function, and where appropriate, provide examples of clinical studies where Fc engineered antibodies have been employed.

## ENHANCED COMPLEMENT-BASED EFFECTOR FUNCTION

Several approved antibodies have demonstrated potent *in vitro* CDC activity such as the anti-CD20 mAbs rituximab and ofatumumab (Teeling et al., 2006), and there are numerous ways that investigators have utilized Fc engineering to enhance complement-based effector function (Table 1). Idusogie et al. demonstrated that K326W/E333S enhanced C1q binding and CDC activity relative to an IgG1, leading the authors to suggest that these two residues play a structural role in interactions between C1q and IgG (Idusogie et al., 2001). Moore et al. demonstrated that the Fc mutations S267E/H268F/S324T enhanced C1q binding 47-fold and CDC activity 6.9-fold over IgG1 (Moore et al., 2010). The authors suggested that the S267E mutant altered the charge of the Fc, potentially increasing ionic interactions with the C1q subunit B. Among the four human IgG subclasses, IgG3 has the most potent CDC activity (Tao et al., 1993). However, there are no approved IgG3 therapeutics due in part to the relatively short half-life of IgG3 as well as bioprocessing issues associated with IgG3’s long hinge region (Brezski and Georgiou, 2016). To overcome the liabilities associated with IgG3, Natsume et al. generated a cross IgG subclass variant combining the CH1 and hinge regions of IgG1 with the CH2 and CH3 regions of IgG3 (Natsume et al., 2008), which resulted in up to 50-fold enhanced CDC activity relative to IgG1 depending on the cell line used to assess CDC activity. This enhanced activity may be attributable to the differences between IgG1 and IgG3 in the CH2 region (i.e., K274Q/N276K/Y300F), which are structurally proximal to the residues D270/K322/P329/P331 deemed critical for C1q interactions (Idusogie et al., 2000; Thommesen et al., 2000). Diebolder et al. employed an approach where they engineered the Fc region with the mutations E345R/E430G/S440Y, which resulted in preformed IgG hexamers (Diebolder et al., 2014) and enhanced CDC activity. Structural data indicated that the hexamer oriented K322 in a position where it could favorably accommodate interactions with the hexameric C1q headpiece. Together, these studies demonstrated that several different mutational approaches can enhance C1q binding and the resultant CDC activity.

**Table 1:** Examples of modifications to modulate antibody effector function. **Unless otherwise noted, the mutations are on the IgG1 subclass.**

Engineering and intended function	Mutation	Reference
**Enhance ADCC**		
Increased FcγRIIIa binding	F243L/R292P/Y300L/V305I/P396L	(Stavenhagen et al., 2007)
Increased FcγRIIIa binding	S239D/I332E	(Lazar et al., 2006)
Increased FcγRIIIa binding, Decreased FcγRIIb binding	S239D/I332E/A330L	(Lazar et al., 2006)
Increased FcγRIIIa binding	S298A/E333A/K334A	(Shields et al., 2001)
	In one heavy chain: L234Y/L235Q/G236W/S239M/H268D/D270E/S298A In the opposing heavy chain: D270E/K326D/A330M/K334E	(Mimoto et al., 2013a)
**Enhance ADCP**		
Increased FcγRIIa binding, Increased FcγRIIIa binding	G236A/S239D/I332E	(Richards et al., 2008)
**Enhance CDC**		
Increased C1q binding	K326W/E333S	(Idusogie et al., 2001)
Increased C1q binding	S267E/H268F/S324T	(Moore et al., 2010)
Increased C1q binding	IgG1/IgG3 cross subclass	(Natsume et al., 2008)
Hexamerization	E345R/E430G/S440Y	(Diebolder et al., 2014)
**Reduce effector function**		
Aglycosylated	N297A or N297Q or N297G	(Bolt et al., 1993; Leabman et al., 2013; Tao and Morrison, 1989; Walker et al., 1989)
Reduced FcγR and C1q binding	L235E	(Alegre et al., 1992)
Reduced FcγR and C1q binding	IgG1: L234A/L235A; IgG4:F234A/L235A	(Xu et al., 2000)
Reduced FcγR and C1q binding	IgG2/IgG4 cross isotype	(Rother et al., 2007)
Reduced FcγR and C1q binding	IgG2: H268Q/V309L/A330S/P331S	(An et al., 2009)
Reduced FcγR and C1q binding	IgG2: V234A/G237A/P238S/H268A/V309L/ A330S/P331S	(Vafa et al., 2014)
**Increase half-life**		
Increased FcRn binding at pH 6.0	M252Y/S254T/T256E	(Dall’Acqua et al., 2002)
Increased FcRn binding at pH 6.0	M428L/N434S	(Zalevsky et al., 2010)
**Increased coengagement**		
Increased FcγRIIb binding	S267E/L328F	(Chu et al., 2008)
Increased FcγRIIa binding, Decreased FcγRIIIa binding	N325S/L328F	(Shang et al., 2014)

## ENGINEERING FOR FcγR-BASED EFFECTOR FUNCTION

Early studies defining key amino acid binding sites on IgGs for FcγRs were performed by mutational analyses, and it was determined that the lower hinge and proximal CH2 regions were critical (Burton et al., 1988; Canfield and Morrison, 1991; Chappel et al., 1991; Duncan et al., 1988; Hulett et al., 1994; Jefferis et al., 1990; Lund et al., 1991; Partridge et al., 1986; Shields et al., 2001; Tamm et al., 1996; Wines et al., 2000; Woof et al., 1986). Additionally, multiple co-crystal structures of human IgG1 Fc with the low affinity FcγRs have allowed high resolution mapping of the binding interfaces. A recurrent finding among the low affinity FcγRs was that P329 packs between two conserved tryptophan residues found in all of the FcγRs (Mimoto et al., 2013b; Radaev et al., 2001; Ramsland et al., 2011; Sondermann et al., 2000)—an interaction termed the “proline sandwich”. More recently, there have been several reports of human IgG1 Fc co-crystals with the high affinity FcγRI (Kiyoshi et al., 2015; Lu et al., 2015; Oganesyan et al., 2015). A common feature among these structures was that a hydrophobic pocket on the surface of FcγRI well-accommodated L235 on the Fc. Together, these mutational studies and crystal structures have resulted in a wealth of knowledge about how IgGs interact with their respective FcγRs.

The efficacy of several anti-cancer mAbs is thought to rely, in part, on recruitment of FcγR-based effector functions. This includes activation of NK cells via FcγRIIIa and resultant ADCC and inflammatory cytokine release, macrophage-mediated ADCP through interactions with multiple FcγRs, and recruitment and activation of other immune cells (e.g., neutrophils). For FcγRIIIa, the key receptor for NK cell-mediated ADCC, there are two polymorphic variants: V158 with higher affinity for IgG1, and F158 that has lower affinity for IgG1. The potential significance of these polymorphisms was reported in several clinical trials where cancer patients with the high-affinity V158 polymorphism showed better outcomes from cetuximab (Bibeau et al., 2009), trastuzumab (Gavin et al., 2017), and rituximab (Cartron et al., 2002) therapy compared to patients with the low-affinity F158 polymorphism, although there were also clinical trials where there was no apparent benefit from expressing the high affinity polymorphism. Due to the potential benefits of augmenting binding to FcγRs and the resultant enhanced innate immune cell function, investigators have utilized multiple approaches to engineer mAbs including glyco-engineering and mutating amino acids within the Fc region.

### Glyco-engineering

IgGs contain a conserved glycosylation site at amino acid N297 in the CH2 domain. The core structure of the glycan is comprised of *N-*acetylglucosamine (GlcNAc) and mannose, where additional modifications can include bisecting GlcNAc, fucose, galactose, and sialic acid. One of the first reports linking glyco-engineering with enhanced Fc effector function demonstrated that IgG1 antibodies produced in a Chinese hamster ovary (CHO) cell line expressing b (1,4)-N-acetylglucosaminyltransferase III to express bisecting GlcNAc augmented ADCC activity relative to IgG1 (Umana et al., 1999). A study by Shields et al. demonstrated that IgG1s deficient in fucose had an up to 50-fold increase in FcγRIIIa binding relative to IgG1 as well as enhanced ADCC (Shields et al., 2002). Shinkawa et al. later demonstrated that fucose deficient antibodies had improved ADCC function compared to antibodies containing bisecting GlcNAc (Shinkawa et al., 2003). The putative mechanism for enhanced interactions between afucosylated antibodies and FcγRIIIa was shown in a crystallographic study (Ferrara et al., 2011). Amino acid N162 in FcγRIIIa contains a glycan, and the absence of fucose allows greater carbohydrate-carbohydrate interactions with the Fc, which increases the overall binding strength. At present, there are two glyco-engineered antibodies that have been approved, the anti-CD20 mAb, obinutuzumab, and the anti-CCR4 mAb, mogamulizumab (Beck and Reichert, 2012). These two antibodies demonstrate that glyco-engineering for enhanced effector function can translate into clinically approved therapeutics.

### Amino acid mutations

There are several ways in which investigators have mutated the amino acid sequence of the IgG Fc region to modulate FcγR-based effector function. Some examples include point mutations, design algorithms, yeast display, and asymmetric engineering. The results from each of these different technologies have yielded numerous mutations that modify Fc-FcγR interactions and the resultant effector functions (Table 1).

Shields et al. performed single alanine point mutations of all solvent-exposed amino acids in CH2 and CH3 domains of human IgG1. They further demonstrated that combining several identified mutations (e.g., S298A/E333A/K334A) resulted in enhanced ADCC relative to IgG1 (Shields et al., 2001). Lazar et al. engineered a series of Fc variants with optimized Fcγ receptor affinity using computational design algorithms and high-throughput screening. The authors identified S239D/I332E and S239D/I332E/A330L as two variants with enhanced ADCC activity (Lazar et al., 2006). A crystal structure of an Fc fragment with the mutations S239D/A330L/I332E was solved and modeling studies suggested that additional hydrogen bonds, hydrophobic contacts, and/or electrostatic interactions resulted in enhanced binding to FcγRIIIa (Oganesyan et al., 2008). Richards demonstrated that the addition of G236A to the S239D/I332E mutations resulted in up to 70-fold improved binding to FcγRIIa, a 13-fold improvement in the FcγRIIa/FcγRIIb binding ratio (activating/inhibitory ratio), and enhanced phagocytosis of antibody-coated target cells by macrophages (Richards et al., 2008). Stavenhagen et al. utilized yeast surface display to identify the variant F243L/R292P/Y300L/V305I/P396L, which showed >100 fold increased ADCC activity (Stavenhagen et al., 2007). Margetuximab, an anti-HER2 mAb with these 5 mutations completed a phase I clinical trial for patients with HER2-overexpressing carcinomas (Bang et al., 2017). Mimoto et al. designed antibody variants with an asymmetrically engineered Fc domain by introducing different amino acid changes in each Fc domain. They screened ~1000 variants and demonstrated that L234Y/L235Q/G236W/S239M/H268D/D270E/S298A changes in one Fc domain and D270E/K326D/A330M/K334E changes in the other increased affinity for FcγRIIIa F158 by more than 2000-fold and FcγRIIIa V158 by more than 1000-fold (Mimoto et al., 2013a). Together, these investigations demonstrate that various technologies can be employed to obtain Fc variants with enhanced FcγR-dependent effector function.

## Fc ENGINEERING FOR REDUCED EFFECTOR FUNCTION

For cases where mAbs are intended to engage cell surface receptors and prevent receptor-ligand interactions (i.e., antagonists), it may be desirable to reduce or eliminate effector function for example to prevent target cell death or unwanted cytokine secretion. Other examples where reduced effector function may be warranted include preventing antibody-drug conjugates from interacting with FcγRs leading to off-target cytotoxicity (Uppal et al., 2015). The need for reducing or eliminating effector function was recognized with the first approved mAb, the murine IgG2a anti-CD3ε mAb, OKT3, muromonab, which was intended to prevent T cell activation in tissue transplant patients receiving a donor kidney, lung, or heart (Chatenoud and Bluestone, 2007). Many patients receiving muromonab had adverse events including the induction of pro-inflammatory cytokines (i.e., cytokine storm), which was attributed in part to muromonab’s interactions with FcγRs (Alegre et al., 1992). In order to reduce this unintended effector function, Bluestone and colleagues generated the human IgG4 variant L235E (Alegre et al., 1992) or F234A/L235A (Xu et al., 2000), and the human IgG1 variant L234A/L235A (Xu et al., 2000), all of which reduced inflammatory cytokine release.

Another early approach intended to reduce effector function was to mutate the glycosylation site at N297 with mutations such as N297A, N297Q, and N297G (Bolt et al., 1993; Leabman et al., 2013; Tao and Morrison, 1989; Walker et al., 1989). This aglycosylation approach has proven successful in abrogating Fc interactions with the low affinity FcγRs and effector functions such as CDC and ADCC. However, it was recognized early on that under avidity-based binding conditions, afucosylated IgGs have been shown to retain effector function when monocytes were employed as effector cells (Bolt et al., 1993). More recently, it was demonstrated that aglycosylated IgG1 mAbs had macrophage-mediated ADCP similar to IgG1 in the absence of competing serum via interactions with FcγRI (Lo et al., 2017; Nesspor et al., 2012). However, in the presence of 10% serum, the aglycosylated IgG1 had undetectable ADCP activity, suggesting that aglycosylated IgGs would have reduced effector function in circulation, which has serum IgG levels ranging from 10 to 20 mg/mL (Nesspor et al., 2012).

Among the four IgG subclasses, each has a different ability to elicit immune effector functions. For instance, IgG1 and IgG3 have been recognized to recruit complement more effectively than IgG2 and IgG4 (Tao et al., 1993). Additionally, IgG2 and IgG4 have very limited ability to elicit ADCC (Brezski et al., 2014). Therefore, several investigators have employed a cross-subclass approach to reduce effector function. The approved anti-C5 therapeutic, eculizumab, has the IgG2 amino acids 118 to 260 and the IgG4 amino acids 261 to 447 and has been shown to have limited or undetectable effector function (Rother et al., 2007). As such, eculizumab represents the first proof-of-concept that reduced effector function mAbs can be safely utilized in an approved mAb-based therapeutic. In a further refinement of the cross-subclass approach, An et al. generated an IgG2 variant with point mutations from IgG4 (i.e., H268Q/V309L/A330S/P331S). This variant had reduced effector function as well as a circulating half-life comparable to that of IgG1 in rhesus monkeys (An et al., 2009). In a similar approach, a variant was reported that contained the IgG2 to IgG4 cross-subclass mutations V309L/A330S/P331S combined with the non-germline mutations V234A/G237A/P238S/H268A, which resulted in undetectable CDC, ADCC, and ADCP (Vafa et al., 2014). The crystal structure of the mutated Fc was solved and modeling studies suggested that conformational changes at P329 would not be favorable for formation of the “proline sandwich” and changes at D270 could negatively impact C1q interactions. Together, these studies demonstrate the numerous approaches that can be taken to reduce or eliminate effector function in hope of improving the safety of mAb therapeutics.

## Fc ENGINEERING FOR COENGAGEMENT OF ANTIGEN AND FcγRS

There are some cases where a potential therapeutic mAb’s target antigen is found on cells that also express FcγRs. Chu et al. engineered a variant to capitalize on such a situation by augmenting binding to FcγRIIb, the primary Fc receptor expressed on B cells. The variant S267E/L328F has greater than 400-fold increased binding to FcγRIIb compared to IgG1 (Chu et al., 2008). Szili et al. showed that coengagement of CD19 and FcγRIIb with a mAb containing the S267E/L328F variant resulted in FcγRIIb ITIM phosphorylation, recruitment of SH2 domain-containing inositol polyphosphate 5-phosphatase (SHIP), and inhibition of downstream signaling pathways in B cells (e.g., Akt) (Szili et al., 2014). In contrast to utilizing coengagement of antigen with the inhibitory FcγRIIb, there are situations where antigen and FcγR coengagement can dramatically increase mAb potency through avidity-based interactions. Loyau et al. showed that a full length IgG1 anti-TLR4 mAb was 1000-fold more potent than the F(ab′)_2_ fragment version (Loyau et al., 2014). This was attributable to the Fab arms engaging TLR4 while the Fc simultaneously engaged FcγRI on the same cell. Coengagement of TLR4 and FcγRIIa resulted in increased potency (i.e., half maximal inhibitory concentration), but to a lesser effect (~25-fold). The antibody was engineered with two Fc mutations, N325S/L328F, which enhanced binding to FcγRIIa (Shang et al., 2014). The Fc engineered anti-TLR4 mAb has completed phase I clinical trials (Monnet et al., 2017).

One consideration for engineering mAbs for coengagement of antigen with FcγRs is the induction of FcγR signaling pathways. For the S267E/L328F variant, engagement of FcγRIIb ITIM signaling was intended (Chu et al., 2008). However, for other receptors such as FcγRIIa, not only is there the potential to engage ITAM signaling, there is also the possibility to engage inhibitory ITAM (ITAMi) signaling—an event where instead of recruiting kinases, the ITAM recruits phosphatases (Blank et al., 2009). Shang et al. tested if the anti-TLR4 mAb engaged ITAMi signaling and showed that there was no activation of Src homology 2 domain containing protein tyrosine phosphatase (SHP-1) (Shang et al., 2014). Novel cases where coengagement is employed should assess downstream ITIM, ITAM, and ITAMi signaling pathways to determine whether or not the antibodies engage unintended signaling events.

## FcRn ENGINEERING TO ALTER HALF-LIFE

The neonatal Fc receptor (FcRn) is a MH1Like heterodimer consisting of a major histocompatibility MH1Like alpha chain non-covalently associated with beta-2-microglobulin (β2m) (Burmeister et al., 1994a) that plays a central role in the cellular trafficking and serum half-life of IgGs (Rath et al., 2013). Its name originates from historical observations leading to the search for a receptor mediating the transfer of humoral immunity from mother to neonatal rats (Brambell, 1966). Research in the early 1960s supported a mechanism of IgG transport specifically involving the Fc region (Brambell, 1966; Brambell et al., 1964; Fahey and Robinson, 1963). It was postulated that a single receptor might control both the transport of IgG during early life and the protection of IgG from catabolism later in life (Brambell et al., 1964). These studies led to the current understanding that the long circulating half-life of antibodies (7–21 days depending on the IgG subclass (Morell et al., 1970)) is attributed, in large part, to their ability to bind FcRn through the Fc. The role of FcRn in IgG homeostasis was further supported by genetic studies where β2m knockout mice had lower levels of circulating IgG (Ghetie et al., 1996; Israel et al., 1996; Junghans and Anderson, 1996).

FcRn is primarily expressed within endosomes where it is capable of binding to IgG internalized through pinocytosis. Conserved histidine residues in the CH2-CH3 domains of IgG become protonated at acidic endosomal pH (6.0–6.5), driving pH-dependent binding to the α-chain of FcRn. Subsequent recycling and release of IgG to the bloodstream at physiological pH (7.4) salvages the antibody from lysosomal degradation (Roopenian and Akilesh, 2007). Mutational and x-ray crystallographic analyses provided insights into the amino acids involved in the Fc:FcRn interaction (Burmeister et al., 1994b; Kim et al., 1999; Martin et al., 2001; Medesan et al., 1997). More recently, the first co-crystal structure of an engineered human Fc with human FcRn provided a molecular understanding of Fc:FcRn interactions (Oganesyan et al., 2014). Key contact positions on the Fc region with FcRn include L251, M252Y (one of the Fc mutations), I253, L309, H310, L314, Q311, and N434 (Fig. 1 and Table 2). The authors further implicated strong hydrogen bonding through H310 as critical for pH dependent binding. Indeed, mutation of this amino acid to any other amino acid (excluding cysteine) resulted in undetectable binding to FcRn at pH 6.0. These characterizations of Fc:FcRn interactions combined with understanding pH dependent Fc:FcRn interactions ignited interest in modulating the pharmacokinetic (PK) properties of therapeutic antibodies through FcRn-mediated recycling mechanisms. Mutations at various positions proximal to the CH2-CH3 interface have been engineered with the intent that prolonged serum half-life may benefit patients by lowering the therapeutic dose and/or frequency of administration and reducing the cost of care (Table 1).

**Table 2:**
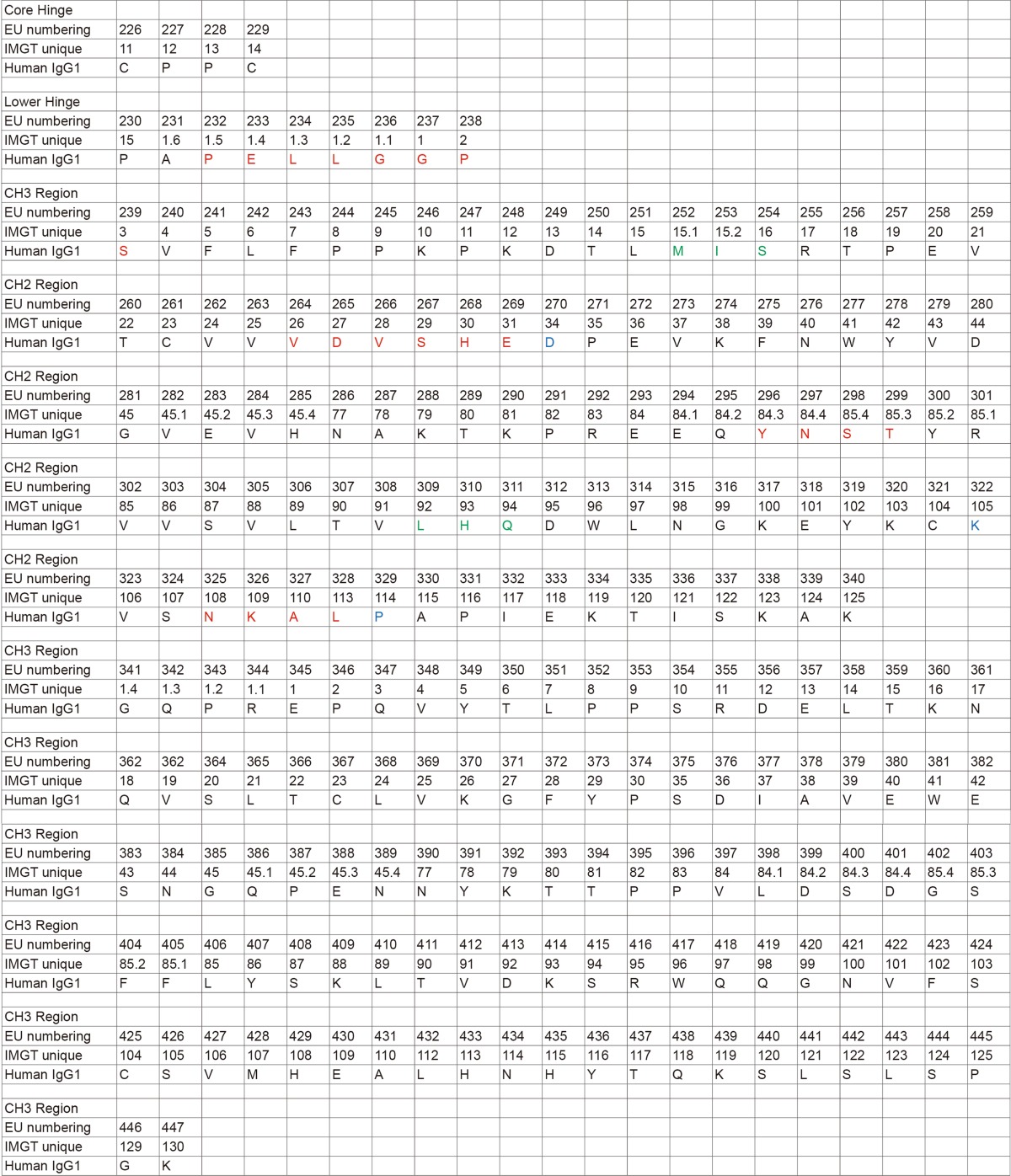
An alignment of the hinge, CH2, and CH3 region for human IgG1 according to IMGT unique numbering (http://www.imgt.org/IMGTScientificChart/Numbering/Hu_IGHGnber.html) **and EU numbering is shown.**

Note: The core hinge region is defined by the N-terminal and C-terminal cysteines that link the two heavy chains together (Liu and May, 2012). The lower hinge region ends with the final amino acid prior to the start of the first CH2 β-sheet domain (as shown in PDBID: 3AVE, 1HZH, 5KD3, and 4HAG). The red, green, and blue coloring scheme is the same that was used in Fig. 1

Screening of a phage display Fc variant library identified mutations (e.g., M252Y/S254T/T256E—termed YTE) with enhanced binding to human FcRn at pH 6.0 (Dall’Acqua et al., 2002). A later study demonstrated that the YTE variant had 10-fold increased binding to human and cynomolgus FcRn at pH 6.0, which translated into a 4-fold increase in half-life in a cynomolgus PK study (Dall’Acqua et al., 2006). Crystal structure analysis indicated that the T256E mutation provided 2 novel salt bridges between the Fc and β2m subunit of FcRn (Oganesyan et al., 2014). In another report, Zalesky et al. showed that the mutations M428L/N434S resulted in an 11-fold increase in affinity for human FcRn at pH 6.0 (Zalevsky et al., 2010). When human FcRn transgenic tumor-bearing mice were treated with either an anti-EGFR antibody or an anti-VEGF antibody containing the M428L/N434S mutations, an increased reduction in tumor burden was observed compared with IgG1 treated animals (Zalevsky et al., 2010). This was the first *in vivo* demonstration of improved anti-tumor activity resulting from an FcRn-dependent increase in half-life. It was postulated that the N434S mutation allowed additional hydrogen bonds with FcRn (Oganesyan et al., 2014), resulting in the increased binding. Overall, these cases demonstrated that increasing the affinity of IgG for FcRn at pH 6.0 resulted in extended half-life and efficacy in pre-clinical studies.

Motavizumab-YTE, a humanized antibody targeting respiratory syncytial virus, is the first antibody engineered for FcRn mediated half-life extension to be tested in human subjects (Robbie et al., 2013). The results from this phase I clinical trial in healthy adults demonstrated a 2- to 4-fold increase in half-life relative to motavizumab IgG1 depending on the dose, which provided proof-of-concept that FcRn low pH enhancing modifications translated into increased half-life in humans.

## CONCLUSIONS

As there are currently 70 phase III and 575 phase I/II antibody-based drugs in clinical trials (Strohl, 2017), competition for targets is increasing, and the need to differentiate antibodies is becoming imperative. The successful approval of the effector function modulated mAbs eculizumab, obinutuzumab, and mogamulizumab has demonstrated that altering effector function can be a clinically viable way to differentiate mAbs from standard human IgG subclasses. With an increasing number of Fc engineered mAbs progressing through clinical trials, it will be interesting to see if modulating antibody effector function leads to the next generation of approved mAb-based therapeutics.
